# A simple, fast and inexpensive approach using *E. coli* to detect and estimate vitamin B_12_ content in microbial extracts

**DOI:** 10.1242/bio.062017

**Published:** 2025-09-03

**Authors:** Katarzyna Hencel, Matthew J. Sullivan, Alper Akay

**Affiliations:** ^1^ School of Biological Sciences, University of East Anglia, Norwich, NR4 7TJ, UK; ^2^ Centre for Microbial Interactions, Norwich Research Park, Norwich, NR4 7UJ, UK

**Keywords:** Vitamin B_12_, Cobalamin, *Caenorhabditis elegans*, *C. elegans*, *E. coli*, MetE, CeMbio

## Abstract

Vitamin B_12_ is an essential micronutrient produced only by prokaryotes, and animals must acquire it from their diet. Vitamin B_12_ is critical for the synthesis of methionine and propionyl-CoA metabolism. In humans, vitamin B_12_ deficiency has been linked to many disorders, including infertility and developmental abnormalities. The growing trend towards plant-based diets and ageing populations increases the risk of vitamin B_12_ deficiency, and, therefore, there is an increasing interest in understanding vitamin B_12_ biology. Accurate approaches for detecting and quantifying vitamin B_12_ are essential in studying its complex biology, from its biogenesis in Bacteria and Archaea to its effects in complex organisms. Here, we present an approach using the commonly available *E. coli* methionine auxotroph strain B834 (DE3) and a multi-well spectrophotometer to detect and estimate the levels of vitamin B_12_ from biological samples at picomolar concentrations. We further show that our method is sufficient to reveal important differences in the production of vitamin B_12_ from vitamin B_12_-synthesising bacteria commonly found in the microbiome of wild *Caenorhabditis elegans* isolates. Our results establish a high-throughput and simple assay platform for detecting and estimating vitamin B_12_ levels using the *E. coli* B834 (DE3) strain.

## Teaser

**Summary:** *E. coli* methionine auxotroph strain B834 (DE3) and a multi-well spectrophotometer can be used to detect and estimate the levels of vitamin B_12_ from biological samples at picomolar concentrations.

## INTRODUCTION

Cobalamin (the natural form of vitamin B_12_) is, structurally, the most complex vitamin. It is only produced by bacteria and archaea and requires more than a dozen enzymes for its biogenesis. In many organisms, cobalamin (vitamin B_12_ hereafter) is essential for the function of two critical enzymes: methionine synthase, which regenerates methionine in the cells from homocysteine, and methylmalonyl-CoA mutase, which converts propionyl-CoA into succinyl-CoA. In humans, vitamin B_12_ deficiency has been linked to multiple diseases, including anaemia, infertility, and developmental and neurological disorders (Mischoulon et al., 2000; Molloy et al., 2008). Although clinical levels of vitamin B_12_ deficiency are rare (Green et al., 2017), the global increase in plant-based diets and ageing populations are linked to reduced vitamin B_12_ uptake, which is considered a growing global health risk that necessitates further molecular and medical research into vitamin B_12_ and its roles in human and animal physiology (Brouwer-Brolsma et al., 2015; Niklewicz et al., 2023).

Research on vitamin B_12_ requires sensitive detection and quantification methods. In particular, previous studies in *Caenorhabditis elegans* have shown that vitamin B_12_ affects animal development and fecundity in a dose-dependent manner (Bito et al., 2017, 2013; Watson et al., 2016). Therefore, accurate measurement of vitamin B_12_ levels in animal diets would facilitate better analysis of the link between vitamin B_12_ and development. Using vitamin B_12_ auxotrophy in bacteria to quantify vitamin B_12_ in biological samples has been a standard method since the 1940s when *Lactobacillus leichamnnii* was described by multiple groups as a suitable strain for vitamin B_12_ quantification (Hoffmann et al., 1949, 1948; Kelleher and Broin, 1991; Skeggs et al., 1948). The assay has been used to this day and is readily available through commercial routes. However, *L. leichamnnii* has complicated growing conditions, including its response to thymidine in the absence of vitamin B_12_ (Kitay et al., 1950).

Another bacterial strain commonly used for vitamin B_12_ assays is *Salmonella typhimurium* mutants, which lack the vitamin B_12_-independent methionine synthase, MetE (Raux et al., 1996). However, *Salmonella* strains grow under anaerobic conditions, often requiring additional apparatus. Another well-established assay uses the microalgae *Euglena gracilis var. bacillaris* (Hutner et al., 1949). Although considered more accurate, this assay takes 4 to 7 days to complete and involves complicated growth conditions.

Alternatively, methionine auxotroph strains of *Escherichia coli* can also be used for vitamin B_12_ quantification (Chiao and Peterson, 1953; Davis and Mingioli, 1950; Harrison et al., 1951; Johansson, 1953). The advantages of utilising *E. coli* strains for vitamin B_12_ assays include fast growth, which allows the assay to be performed overnight, and simple growth requirements, which do not involve specialised media preparation. However, there is limited information on the sensitivity and specificity of *E. coli*-based vitamin B_12_ assays.

In addition to microbiological methods, several analytical methods have been developed for detecting and quantifying vitamin B_12_ using liquid chromatography (Wongyai, 2000; Moreno and Salvadó, 2000), reverse-phase high-performance liquid chromatography (Heudi et al., 2006; Campos-Gimnez et al., 2008), and liquid chromatography coupled to mass spectrometry (Gentili et al., 2008; Chamlagain et al., 2015). Although some of these methods offer high sensitivity and lower detection levels, these techniques require high-end instruments and significant expertise in chromatography and mass spectrometry. In addition, the results from analytical approaches correlate well with microbiological assays (Campos-Gimnez et al., 2008).

Here, we present a high-throughput bacteria-based assay for vitamin B_12_ detection and relative quantification using the methionine-auxotroph *E. coli* B834 (DE3) strain. This approach allows for simple and time-efficient detection and relative quantification of vitamin B_12_ content in biological samples. We further utilise the method to estimate the vitamin B_12_ content of different bacterial species commonly found with wild isolates of the nematode *C. elegans*.

## RESULTS

### Analysis of *E. coli* B834 methionine and vitamin B_12_ auxotrophy

**Fig. 1.**
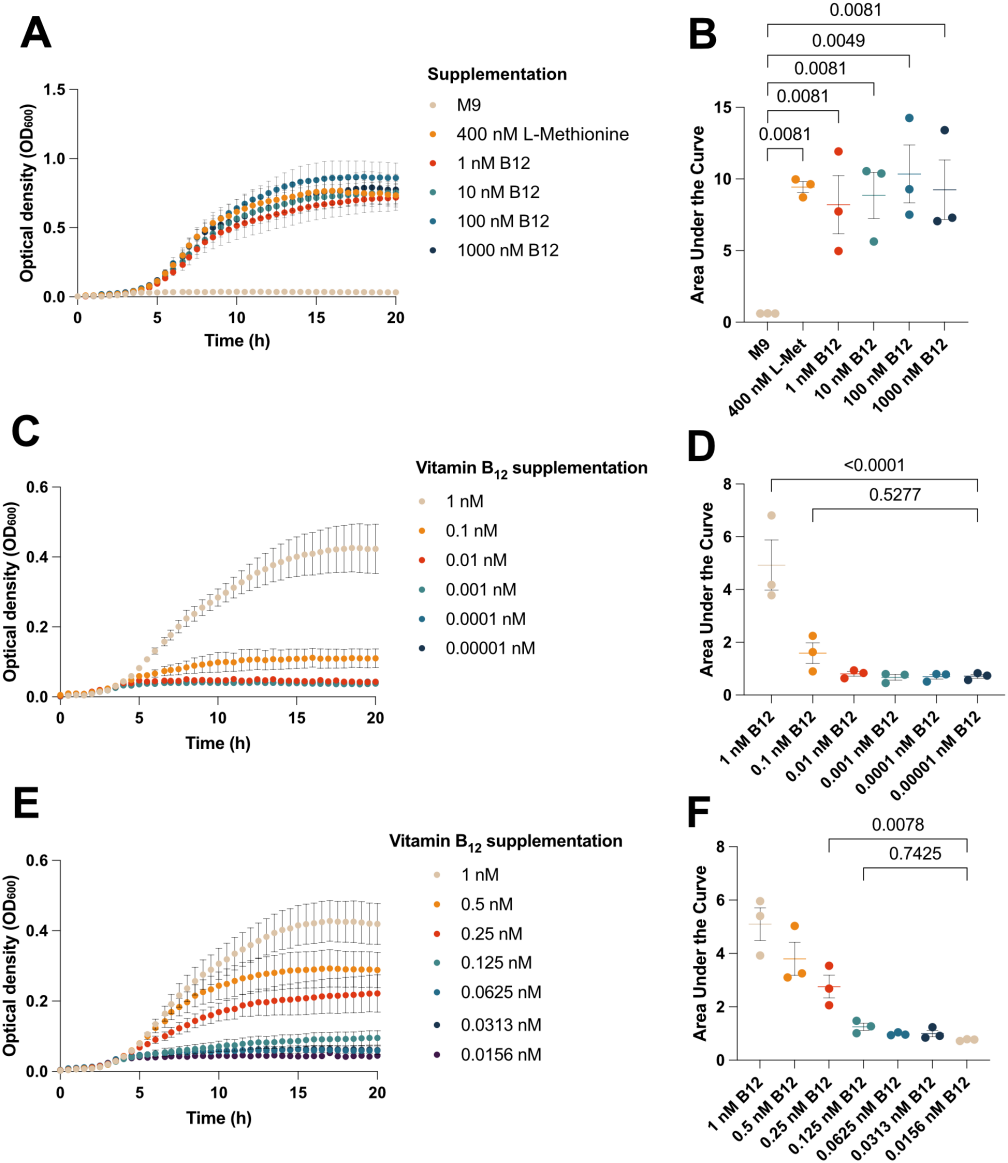
**The growth of *E. coli* B834 (DE3) is dependent on methionine or vitamin B_12_ and is titratable with vitamin B_12_ concentration.** *E. coli* B834 was cultured in M9 minimal media, without or with supplemental methionine or vitamin B_12_ (1 nM–1000 nM) as indicated (A). The growth conditions were compared by AUC analysis, followed by one-way ANOVA with Holm-Šidák multiple comparison corrections and *P*-values indicated (B). *E. coli* B834 growth with vitamin B_12_ supplementation at 1 nM and using 10-fold (C) and 2-fold serial dilutions (E) to test a broad range of sub-nanomolar vitamin B_12_ concentrations. The growth of *E. coli* B834 in the 10-fold and 2-fold were compared by AUC analysis followed by one-way ANOVA and Holm-Šidák multiple comparison corrections and comparisons are shown in D and F, respectively. The data points are plotted as mean±s.e.m. from three biological replicates derived from two technical replicates.

*E. coli* has two methionine synthase enzymes: the B_12_-dependent MetH and the B_12_-independent MetE. *E. coli* B834 (DE3) has a *null* mutation in the *metE* gene, making the bacteria solely dependent on either methionine or vitamin B_12_ supplementation. To assess whether *E. coli* B834 (DE3) is suitable for vitamin B_12_ detection and quantification, we tested the growth of this strain in response to various vitamin B_12_ and methionine supplementations. We confirmed that *E. coli* B834 (DE3) can grow only when methionine or vitamin B_12_ is present in the media (Fig. 1A). We did not observe any significant difference in growth when B_12_ was supplemented at concentrations ranging from 1 nM to 1000 nM, as determined by area under the curve analysis followed by one-way ANOVA with Holm-Šidák multiple comparison corrections (Fig. 1B).

### Using the growth of *E. coli* B834 to estimate vitamin B_12_ levels

**Fig. 2.**
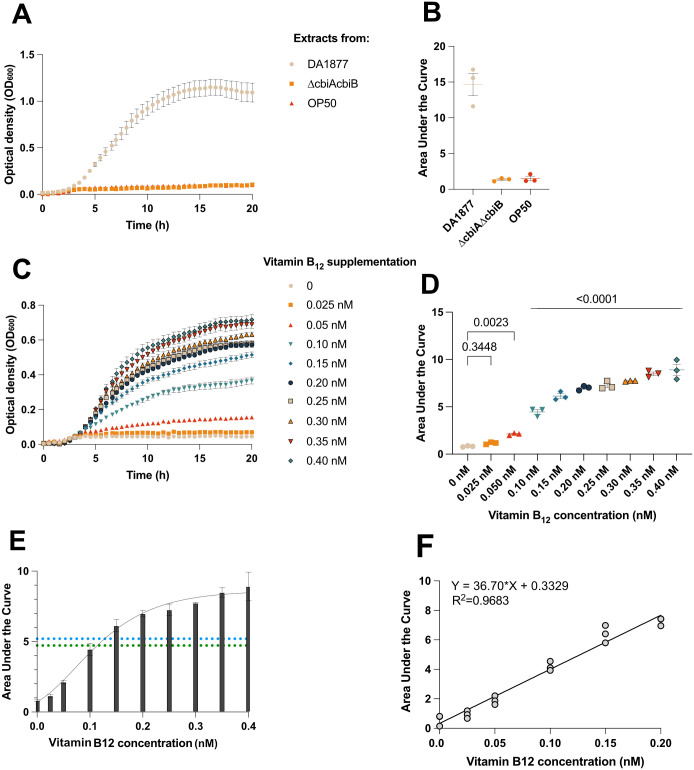
**Use of *E. coli* B834 (DE3) growth to detect and estimate vitamin B_12_ production by *C. aquatica*.** *E. coli* B834 (DE3) was cultured in M9 minimal medium, without or with supplemental vitamin B_12_ at concentrations between 0.025–0.4 nM (A) and compared using AUC analysis coupled with one-way ANOVA with Holm-Šidák multiple comparison corrections (B). *E. coli* B834 culture was supplemented by bacterial cell-free extracts of *C. aquatica* DA1877, Δ*cbiA*Δ*cbiB C. aquatica*, and *E. coli* OP50 (C) and growth in the presence of extracts were compared by AUC analysis coupled with one-way ANOVA with Holm-Šidák multiple comparison corrections (D). AUC data from vitamin B_12_ standards in panel B were used for Gompertz model fitting, further employed for vitamin B_12_ quantification [*Y=YM×(Y0/YM)^(exp(-K×X)*], where YM is the maximum AUC score, Y0 is the minimum AUC score, K determines the lag time (E). The dashed green line corresponds to the 4.70 AUC score of the 10^−1^ dilution of *C. aquatica* DA1877 extract, while the dashed blue line corresponds to the 5.20 AUC score of the 1:8 dilution of *C. aquatica* DA1877 extract, where 2 µl out of 50 µl of the 1 OD extract was used for quantification. In this model, YM=8.606, Y0=0.7062, and K=12.53. (F) Area under the curve analysis of *E. coli* B834 cultures supplemented with vitamin B_12_ concentrations prepared from known standards. The line represents simple linear regression, and the equation and R^2^ value are shown from three independent experiments. All data points are plotted with mean±s.e.m. from three biological replicates derived from two technical replicates.

We subsequently sought to test the utility of using the growth of *E. coli* B834 (DE3) as a highly sensitive biological method for detecting vitamin B_12_. To this end, we used M9 minimal media (devoid of methionine or vitamin B_12_) and added vitamin B_12,_ supplemented at concentrations ranging from 0.00001 nM to 1 nM using 10-fold (Fig. 1C,D) and 2-fold (Fig. 1E,F) serial dilutions. Using this range, we determined that vitamin B_12_ concentrations at and above 0.25 nM (250 pM) were sufficient to support the growth of the *E. coli* B834 (DE3) strain, as evidenced by a significant increase in area under the curve measurements throughout the growth period (Fig. 1F). Next, we prepared a standard curve of vitamin B_12_ concentrations between 0 and 0.4 nM to determine the limit of detection and quantification for vitamin B_12_ using the *E. coli* B834 (DE3) strain. Compared to the unsupplemented media control, the limit of detection is 0.05 nM (50 pM) (Fig. 2A,B), indicating that the growth of *E. coli* was detectable above background absorbance measurements. Increasing the carbon source from 0.4% glucose to 1.0% did not change the sensitivity of the growth assay (Fig. S1A,B). In summary, we have established that the growth of *E. coli* B834 (DE3) can be used to detect vitamin B_12_ at concentrations as low as 50 pM and for relative quantification between concentrations of 50–200 pM.

### Using *E. coli* B834 (DE3) to estimate vitamin B_12_ levels in biological samples

*C. elegans* is a well-established model organism for studying the function of vitamin B_12_ during animal development and for understanding the molecular pathways related to vitamin B_12_ (Bito et al., 2017, 2013, 2019; Bito and Watanabe, 2016; Watson et al., 2014). *C. elegans* exclusively feeds on bacteria, and its uptake of vitamin B_12_ depends on the bacteria available in its environment as a food source. One such bacterium *C. elegans* feeds on in the wild is *Comamonas aquatica* DA1877, a known vitamin B_12_ producer (Watson et al., 2014), which was isolated from soil (Shtonda and Avery, 2006). Mutations in the *cbiA* and *cbiB* genes, which code for cobyrinate a,c-diamide synthase and adenosylcobinamide-phosphate synthase enzymes, respectively, prevent *C. aquatica* from producing vitamin B_12_ (Watson et al., 2014). As a negative control for our assay, we generated an isogenic *ΔcbiAΔcbiB* mutant of DA1877 by deleting the *cbiA* and *cbiB* genes (Fig. S2A–C). To confirm that our *ΔcbiAΔcbiB* strain no longer produced vitamin B_12_, we utilised our *E. coli* B834 (DE3) approach to test for the presence of vitamin B_12_ in cell-free extracts from *C. aquatica.* Briefly, bacterial cells were lysed by boiling, and cell-free extracts were added to *E. coli* B834 (DE3) in media devoid of vitamin B_12_ or methionine. Using this approach, we assayed the vitamin B_12_ levels of wild-type *C. aquatica*, *C. aquatica ΔcbiAΔcbiB* and *E. coli* OP50, a different strain of *E. coli* commonly used as laboratory food for *C. elegans* but known to be a vitamin B_12_ non-producer (Watson et al., 2014; Zimmermann et al., 2020). As predicted, cell-free extracts of wild-type *C. aquatica* DA1877 supported vitamin B_12_-dependent growth of *E. coli* B834 (DE3), whereas the *C. aquatica ΔcbiAΔcbiB* mutant and *E. coli* OP50 did not (Fig. 2C,D). We further estimated the vitamin B_12_ content in *C. aquatica* extracts by using 2-fold and 10-fold serial dilutions and comparing the relative growth of *E. coli* B834 (DE3) with *C. aquatica* extracts against growth with known concentrations of vitamin B_12_ (Fig. S2D,E). Using the 2-fold and 10-fold dilutions combined with either the Gompertz-modelled Area under the Curve analysis (Fig. 2E) or the linear regression model (Fig. 2F) of the standard curve, we estimate the vitamin B_12_ content of *C. aquatica* DA1877 to be approximately 25 nM and 27 nM per 1 OD unit (1 ml of culture at 1.0 OD_600nm_) of bacteria, respectively.

**Fig. 3.**
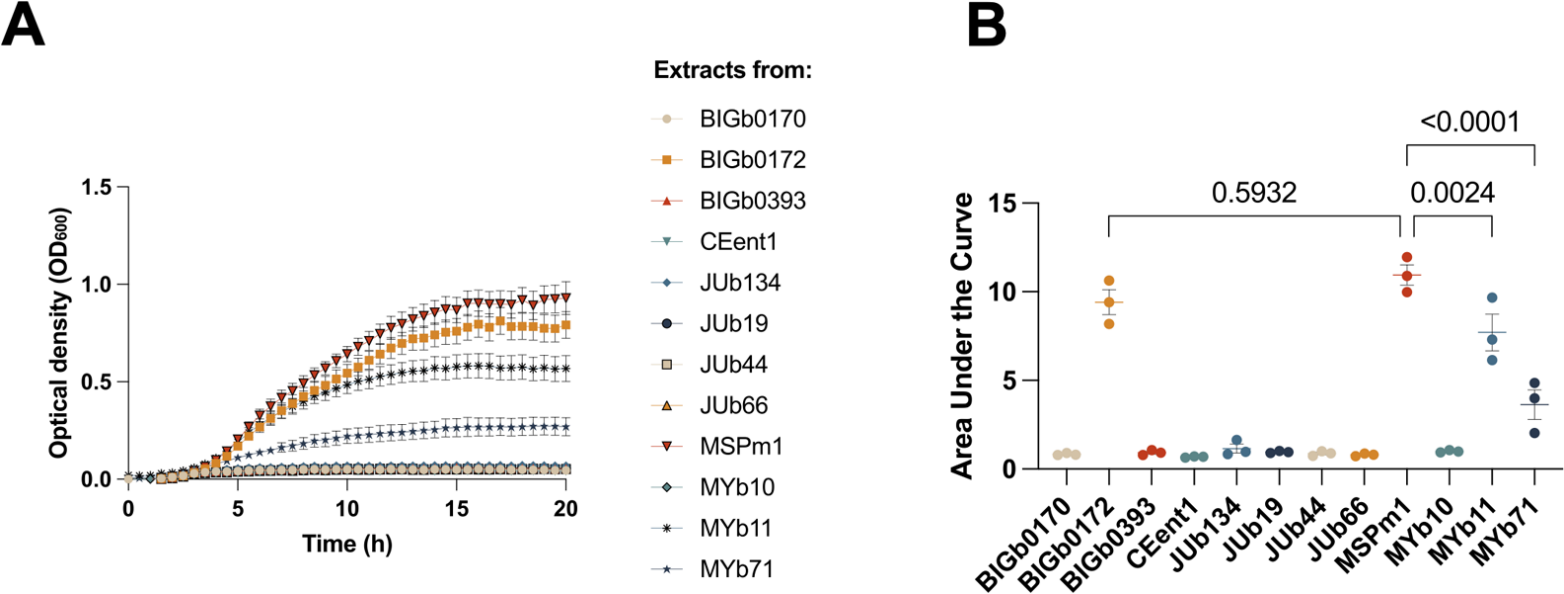
**Detection and estimation of vitamin B_12_ levels in bacterial isolates from wild *C. elegans.*** *E. coli* B834 (DE3) was cultured in M9 minimal media and supplemented with cell-free bacterial extracts of the indicated strains (A). Growth was compared using AUC analysis coupled with one-way ANOVA with Holm-Šidák multiple comparison corrections (B). The data points are plotted with mean±s.e.m. from three biological replicates derived from two technical replicates corrected for blank readings. Bacterial isolates are as follows: *Sphingobacterium multivorum* BIGb0170, *Comamonas piscis* BIGb0172, *Pantoea nemavictus* BIGb0393, *Enterobacter hormaechei* CEent1, *Sphingomonas molluscorum* JUb134, *Stenotrophomonas indicatrix* JUb19, *Chryseobacterium scophthalmum* JUb44, *Lelliottia amnigena* JUb66, *Pseudomonas berkeleyensis* MSPm1, *Acinetobacter guillouiae* MYb10 *Pseudomonas lurida* MYb11, *Ochrobactrum vermis* MYb71.

Our assays with vitamin B_12_, along with extracts from both vitamin B_12_-producing and non-producing bacteria, provided proof of concept for our method to detect vitamin B_12_ in complex biological samples by using the growth of *E. coli* B834 (DE3) as a proxy. Next, we applied this method to assess the vitamin B_12_ content of 12 bacterial strains from the CeMbio collection, all of which were isolated from *C. elegans* found in the wild (Dirksen et al., 2020). Four bacterial strains, *Comamonas piscis* BIGb0172, *Pseudomonas berkeleyensis* MSPm1, *Pseudomonas lurida* MYb11 and *Ochrobactrum vermis* MYb71, were predicted to produce vitamin B_12_ based on their genomic sequences and predicted metabolic pathway analyses (Zimmermann et al., 2020; Dirksen et al., 2020). Our analysis using the *E. coli* B834 (DE3) growth assay showed that *C. piscis* BIGb0172, *P. berkeleyensis* MSPm1, *P. lurida* MYb11, and *O. vermis* MYb71 are indeed vitamin B_12_ producers, because cell-free extracts from cultures of these bacteria were capable of supporting the growth of *E. coli* B834 (DE3) in a manner that relied on vitamin B_12_ (Fig. 3A,B). Among these, extracts from *C. piscis* BIGb0172 and *P. berkeleyensis* MSPm1 supported the highest growth, while *O. vermis* MYb71 showed reduced growth, indicating that there may be variation in the amount of vitamin B_12_ produced by these bacteria. In contrast, supplementing *E. coli* B834 (DE3) with extracts from the other eight CeMbio strains led to a complete absence of bacterial growth (Fig. 3A,B).

In summary, we show that our application of *E. coli* B834 (DE3) growth in minimal media can be used for rapid and high-throughput detection and estimation of vitamin B_12_ levels in biological samples.

## DISCUSSION

Vitamin B_12_-dependent microorganisms are commonly used to detect and quantify vitamin B_12_ in various formats (Hoffmann et al., 1949, 1948; Skeggs et al., 1948). Using *E. coli metE* mutants for this purpose offers numerous advantages, including their commercial availability, rapid growth, and simple growth requirements. However, there is limited information on the sensitivity and reproducibility of *E. coli metE*-based vitamin B_12_ assays. Here, we present a vitamin B_12_ quantification assay using a readily available commercial strain of *E. coli* B834 (DE3) and widely used and inexpensive minimal media. The assay was developed in liquid culture using a 96-well plate format and a multi-well plate reader, allowing for reproducible analysis of many biological samples with a sensitivity as low as at 50 pM concentration.

The dependency on the growth of *E. coli* B834 (DE3) due to the presence of vitamin B_12_ can be confounded by the presence of methionine, which may bypass the metabolic bottleneck caused by vitamin B_12_ limitation in *metE*^−^ *E. coli* and could affect the specificity of our assay. However, previous studies conducted on the *E. coli* 113-3 strain, another methionine auxotroph, showed that methionine must be 50,000 times more concentrated than vitamin B_12_ to hinder vitamin B_12_ quantification using the *E. coli* assay, which was significantly higher than the levels found in the mammalian tissues tested (Chiao and Peterson, 1953). Similarly, we did not observe unexpected *E. coli* B834 (DE3) growth supported by extracts derived from known vitamin B_12_ non-producers (Fig. 2C) or CeMbio collection strains, which were not predicted to produce vitamin B_12_ (Fig. 3). However, we did not test a wider range of culture conditions, such as vitamin B_12_ concentrations above 1 nM, but as highlighted in this work, we predict such levels would be sufficient for growth of the *E. coli* strain used in the present study. In addition, we have not performed a side-by-side comparison with other bacterial isolates used previously for microbiological assays of vitamin B_12_. Therefore, in future work, we recommend additional testing of culture conditions and using a known vitamin B_12_-deficient strain as controls. In addition, the range of relative quantification is between 50 and 200 pM, which is small and should be used with caution, noting that samples may require dilution to be within this range. For absolute quantification of vitamin B_12_, analytical methods using liquid chromatography and mass spectrometry should be considered as the validated approach.

Previous studies suggested that four strains in the CeMbio collection, *C. piscis* BIGb0172, *P. berkeleyensis* MSPm1, *P. lurida*, and *O. vermis* Myb71, are vitamin B_12_ producers, based on the presence of vitamin B_12_ biosynthetic pathway genes (Zimmermann et al., 2020). Our analysis provided experimental evidence to support this. Interestingly, despite confirming that all four isolates are vitamin B_12_ producers, we note that the levels of vitamin B_12_ likely vary significantly, with *P. berkeleyensis* MSPm1 and *C. piscis* BIGb0172 producing significantly higher levels of vitamin B_12_ compared to *P. lurida* Myb11 and *O. vermis* Myb71. These differences in vitamin B_12_ content could be important for the growth of *C. elegans* and other organisms that directly rely on bacteria for vitamin B_12_.

In conclusion, our results establish a high-throughput, straightforward, and cost-effective method for detecting and estimating vitamin B_12_ levels in biological samples. The simplicity, reproducibility, and sensitivity of the *E. coli* B834 (DE3) assay provide an important methodology for the research community working on vitamin B_12_. Our discovery of varying vitamin B_12_ levels in the wild *C. elegans* microbiome makes a compelling case for further investigation into how differences in bacterial metabolites impact animal development. Finally, we recognise that, as we have shown in the present study, growth-based approaches using *E. coli* may be applied to measure other metabolites of interest in a manner that is inexpensive and high-throughput; this area of microbiology should not be forgotten as a powerful functional approach in the biosciences.

## MATERIALS AND METHODS

### Bacterial strains, plasmids and culture media

All bacteria and plasmids are listed in Tables 1 and 2, respectively. Growth media used were M9 minimal salts medium (KH_2_PO_4_, 15 g/l NaCl, 2.5 g/l Na_2_HPO_4_, 33.9 g/l NH_4_Cl, 5 g/l, 2 mM MgSO_4_, 0.1 mM CaCl_2_, 0.4% glucose unless otherwise stated), soya-rich medium (soya peptone 20 g/l, sodium chloride 5 g/l) or Lysogeny broth (LB; 10 g/l NaCl, 10 g/l tryptone, 5 g/l yeast extract). For *E. coli* B834, defective for *metE*, M9 medium was supplemented with 400 nM L-methionine as indicated. *E. coli* OP50, *C. aquatica* DA1877 and the isogenic Δ*cbiA*Δ*cbiB* mutant were grown in the soya-rich medium at 37°C at 180 rpm agitation for the extract preparation. All *E. coli* and *C. aquatica* derivatives were grown at 37°C at 180 rpm agitation, and strains from the CeMbio collection for the extract preparation were grown at 28°C at 180 rpm in the vitamin B_12_-deficient soya-rich medium.

**Table 1.** Bacterial strains used in the study

Strain name	Characteristics	Reference
*E. coli* B834 (DE3)	F^−^ *ompT hsdS*_B_(r_B_^−^ m_B_^−^) *gal dcm met* (DE3)	(Wood, 1966)
*E. coli* OP50	*ura*-, *strR*, *rnc*-, (delta)*attB*::FRT-*lacI*-*lacUV5p*-T7	(Brenner, 1974)
*E. coli* JKE201	RP4-donor for bi-parental conjugation	(Harms et al., 2017)
*Comamonas aquatica* DA1877	Wild-type strain	(Shtonda and Avery, 2006)
ALP121	DA1877-derivative with *cbiB* deletion	This study
ALP122	DA1877-derivative with *cbiA* and *cbiB* deletions	This study
	CeMbio collection	
*Sphingobacterium multivorum* BIGb0170	Wild-type strain	(Dirksen et al., 2020)
*Comamonas piscis* BIGb0172	Wild-type strain	(Dirksen et al., 2020)
*Pantoea nemavictus* BIGb0393	Wild-type strain	(Dirksen et al., 2020)
*Enterobacter hormaechei* CEent1	Wild-type strain	(Dirksen et al., 2020)
*Sphingomonas molluscorum* JUb134	Wild-type strain	(Dirksen et al., 2020)
*Stenotrophomonas indicatrix* JUb19	Wild-type strain	(Dirksen et al., 2020)
*Chryseobacterium scophthalmum* JUb44	Wild-type strain	(Dirksen et al., 2020)
*Lelliottia amnigena* JUb66	Wild-type strain	(Dirksen et al., 2020)
*Pseudomonas berkeleyensis* MSPm1	Wild-type strain	(Dirksen et al., 2020)
*Acinetobacter guillouiae* MYb10	Wild-type strain	(Dirksen et al., 2020)
*Pseudomonas lurida* MYb11	Wild-type strain	(Dirksen et al., 2020)
*Ochrobactrum vermis* MYb71	Wild-type strain	(Dirksen et al., 2020)

**Table 2.** Plasmids used in this study

Plasmid	Description	Reference
pFOK	Suicide vector containing *sacB* and driven by *p_tetA_*_;_ kan^R^	(Cianfanelli et al., 2020)
pAA46	Derivative of pFOK containing up- and downstream fragments of the *cbiB;* kan^R^	This study
pAA47	Derivative of pFOK containing up- and downstream fragments of the *cbiA;* kan^R^	This study

### *C. aquatica* DA1877 vitamin B_12_-deficient mutant generation

Oligonucleotide primers (Table 3) with flanking 20 bp overhangs were designed to amplify upstream and downstream fragments from *cbiA* and *cbiB* of *C. aquatica* DA1877 using Benchling's Gibson Assembly Wizard. The amplified fragments were introduced to the pFOK suicide vector through Gibson Assembly and transformed into *E. coli* JKE201. All constructs were verified by Sanger sequencing, followed by conjugation with *C. aquatica* DA1877 on LB supplemented with 100 µM diaminopimelic acid (DAP) to support *E. coli* JKE201 growth. Transconjugants were selected onto LB agar containing 100 µg/ml kanamycin. At least three transconjugants were grown in LB medium for 4 h, followed by plating on no-salt LB agar plates (10 g/l tryptone, 5 g/l yeast extract, 15 g/l agar) supplemented with 20% sucrose and 0.5 µg/ml anhydro-tetracycline. Candidate colonies were screened for deletions through PCR and verified by Sanger sequencing. Mutation in *cbiB* resulted in the out-of-frame deletion of 178 amino acids, while *cbiA* mutation resulted in complete gene removal. The Sanger sequencing trace files are available as Supplementary File 1.

**Table 3.** Primers used in the study

Primer	5′ – 3′ sequence	Description
A88	TTTCTCTTTGCGCTTGCGTTTCTAGCCCTTATGCAGCCTG	Forward primer for *cbiB* upstream fragment generation
A89	TGGAATGTGGCCGTGCTGTAGCTGAAGATGCGCGAGGAAC	Reverse primer for *cbiB* upstream fragment generation
A90	GTTCCTCGCGCATCTTCAGCTACAGCACGGCCACATTCCA	Forward primer for *cbiB* downstream fragment generation
A91	CGCCAAGCGCGCAATTAACCCGAAGGCTTGCCGCTATCAT	Reverse primer for *cbiB* downstream fragment generation
A92	ATGATAGCGGCAAGCCTTCGGGTTAATTGCGCGCTTGGCG	Forward primer for *cbiB* pFOK backbone generation
A93	CAGGCTGCATAAGGGCTAGAAACGCAAGCGCAAAGAGAAA	Reverse primer for *cbiB* pFOK backbone generation
A101	GAGCCAGATGCGCTACTGAA	Forward primer for *cbiB* mutant screening and validation
A102	TCATGGTGGCTTGAGGCAGC	Reverse primer for *cbiB* mutant screening and validation
A161	TTTCTCTTTGCGCTTGCGTTTCGCCAGCACTTCCAAAAAC	Forward primer for *cbiA* upstream fragment generation
A162	TGGCCCTGGCGGGCACCCCCGACTTCTCCGATGCAACCCT	Reverse primer for *cbiA* upstream fragment generation
A163	AGGGTTGCATCGGAGAAGTCGGGGGTGCCCGCCAGGGCCA	Forward primer for *cbiA* downstream fragment generation
A164	CGCCAAGCGCGCAATTAACCGCGCGTTCAGCGCCACGGCC	Reverse primer for *cbiA* downstream fragment generation
A165	GGCCGTGGCGCTGAACGCGCGGTTAATTGCGCGCTTGGCG	Forward primer for *cbiA* pFOK backbone generation
A166	GTTTTTGGAAGTGCTGGCGAAACGCAAGCGCAAAGAGAAA	Reverse primer for *cbiA* pFOK backbone generation
A338	ACAGCCGGATCATTTGAGCT	Forward primer for *cbiA* mutant screening and validation
A339	CTGTTCCAGCGCTTCTCGCA	Reverse primer for *cbiA* mutant screening and validation

### Bacterial lysate preparation

Bacterial cultures for the *E. coli* B834 (DE3) assay were grown overnight and 1 OD unit (equivalent of 1 ml of culture with absorbance at 600 nm of 1.0) was centrifuged at 15,000 rpm for 1 min. The supernatant was removed, and the cells were resuspended in 50 µl of M9 minimal salts medium and boiled at 100°C for 15 min, as previously described (Ross, 1952). After boiling, lysates were centrifuged at 15,000 rpm for 1 min to remove debris, and the cooled supernatant was used as an extract for supplementation assays.

### *E. coli* B834 (DE3) assay

The assay was prepared in 96-well plates (Greiner #655180) with the final volume of 200 µl of M9 minimal salts medium devoid of methionine unless indicated. Overnight cultures of *E. coli* B834 (DE3) grown in LB were back-diluted 1:100 into the wells and supplemented with either 2 µl of prepared bacterial lysates (extracts) or vitamin B_12_ standard solutions used for the growth curves. The growth response was recorded over 20 h at 37°C with 300 rpm agitation, with readings taken every 30 min using a SPECTROstar*^®^* Nano plate reader (BMG Labtech) in matrix scan mode using a 2×2 scan matrix with 25 flashes per scan point and path length correction of 5.88 mm for 200 µl volume. For blank corrections of optical density readings, control wells containing media without bacteria were included. Methylcobalamin (Thermo Scientific Chemicals, #A11176ME) was used for the vitamin B_12_ standard curve.

### Statistical analysis and visualisation

Data were analysed and visualised using Prism 10 (Version 10.3.0). AUC analysis provides a comprehensive measure of bacterial growth by integrating OD600 readings over time, capturing the full dynamics of the growth curve – including lag, exponential, and stationary phases. Unlike single-point measurements, AUC reflects total biomass accumulation and is less affected by transient fluctuations or noise in the data. With high-resolution measurements taken every 30 min over 20 h, AUC was used as a robust and quantitative way to compare overall growth performance across strains or treatment conditions, especially when differences are subtle or affect growth kinetics rather than final density. To assess statistical significance between groups, we used one-way ANOVA followed by Holm-Šidák multiple comparisons testing, which controls for type I error while maintaining statistical power across multiple pairwise comparisons, with specific details described in the figure legends.

## Supplementary Material



10.1242/biolopen.062017_sup1 Supplementary information
