# Supplementary figures and images for: A simple, fast and inexpensive approach using E. coli to detect and estimate vitamin B12 content in microbial extracts
Source: Biol Open. 2025 Sep 3;14(9):bio062017. doi: 10.1242/bio.062017 (PMC12444877; doi:10.1242/bio.062017)

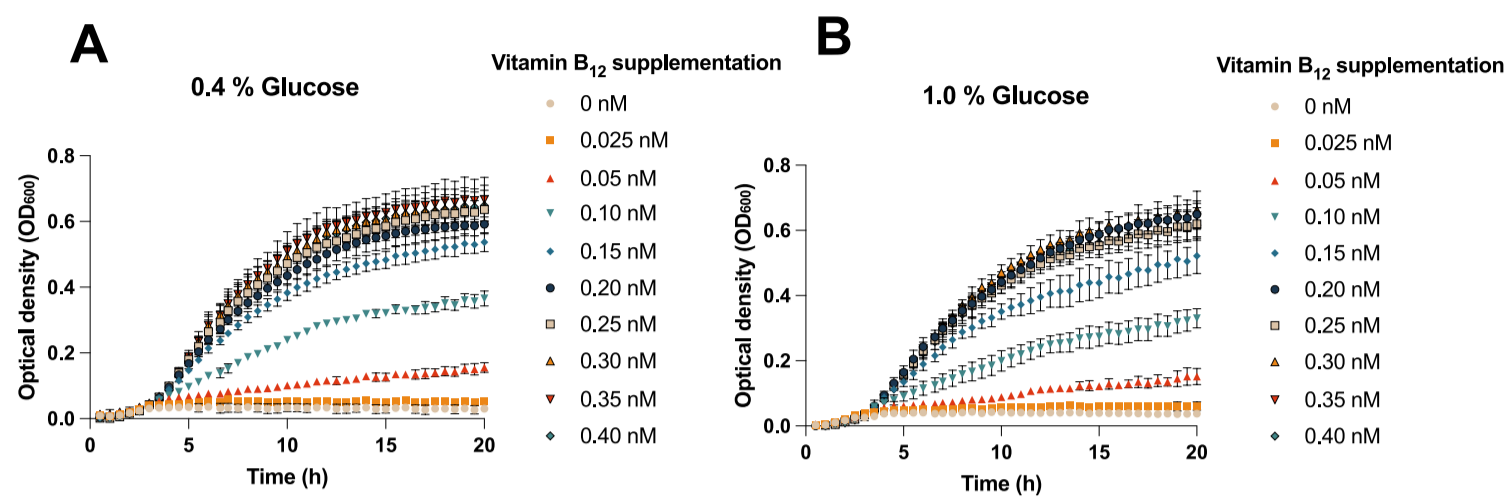

Fig. S1.

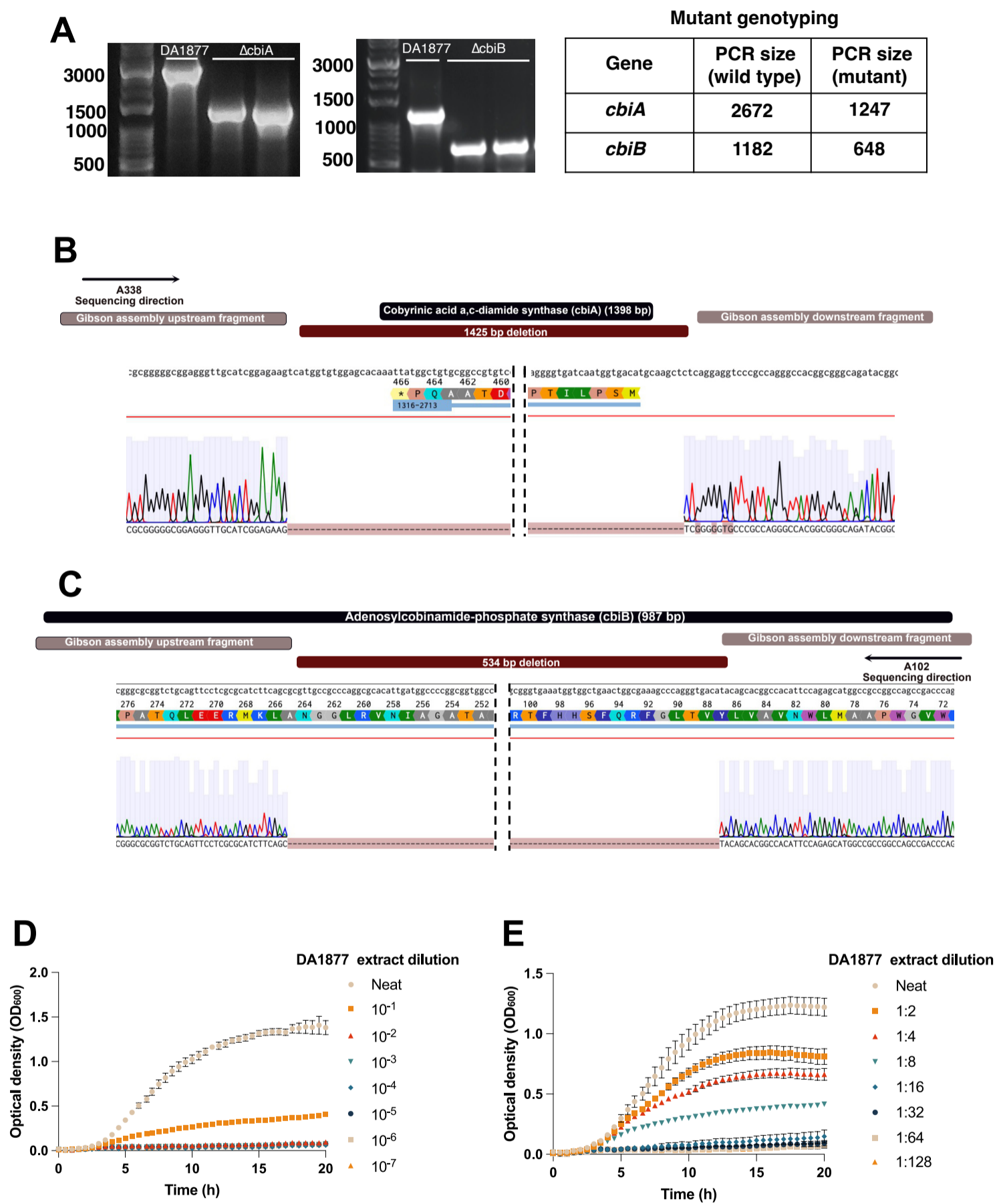

Fig. S2.

Supplement: Supplementary information [file biolopen-14-062017-s1.pdf]
